# Prevalence, risk factors and genetic diversity of *Chlamydia felis* in cats

**DOI:** 10.2478/jvetres-2025-0074

**Published:** 2025-12-23

**Authors:** Monika Szymańska-Czerwińska, Kinga Zaręba-Marchewka, Michał Woś, Ireneusz Balicki, Jowita Zwolska, Barbara Kuduk, Krzysztof Niemczuk

**Affiliations:** Department of Bacteriology and Animal Bacterial Diseases, National Veterinary Research Institute, 24-100 Puławy, Poland; Department of Biotechnology and Nutrigenomics, Institute of Genetics and Animal Biotechnology, Polish Academy of Sciences, 05-552 Magdalenka, Poland; Department of Medical Informatics and Statistics with the e-Health Laboratory, Medical University of Lublin, 20-059 Lublin, Poland; Department and Clinic of Animal Surgery, Faculty of Veterinary Medicine, University of Life Sciences in Lublin, 20-950 Lublin, Poland

**Keywords:** *Chlamydia felis*, cat, chlamydiosis, real-time PCR, risk factors

## Abstract

**Introduction:**

*Chlamydia felis* is the main chlamydial pathogen of cats and is associated with conjunctivitis and respiratory disease. This study aimed to estimate the prevalence of *Chlamydiaceae* and *Chlamydia felis*, to explore risk factors and predictors (age, sex, breed, origin and ocular signs) for infection using logistic regression, and to appraise genetic diversity *via omp*A sequencing and phylogenetic analysis.

**Material and Methods:**

Conjunctival swabs from 156 cats were examined using real-time PCR assays for *Chlamydiaceae* and *C. felis*. Logistic regression and Kaplan–Meier analysis evaluated risk factors, and partial *omp*A sequences were phylogenetically analysed.

**Results:**

*Chlamydiaceae* DNA was detected in 7.7% (12/156) of cats and was identified as *C. felis*. Infections were mostly unilateral. Threshold cycle values varied widely, suggesting heterogeneous bacterial loads. Younger age was a significant risk factor, and the probability of infection decreased steadily with age. Ocular signs strongly predicted infection. British Shorthair/Longhair cats had more than threefold higher odds of infection than European Shorthair cats. Phylogenetic analysis of *omp*A showed very high genome conservation (99.7–100%), which was consistent with global data.

**Conclusion:**

This first molecular study of *C. felis* in Poland in ten years demonstrates that infection mainly affects young, purebred cats with apparent conjunctivitis. The genetic stability of *omp*A supports the concept of a globally homogeneous *C. felis* population.

## Introduction

The Chlamydia genus in the *Chlamydiaceae* family has long been known to cause infections in both animals and humans. Currently, multiple species and several candidate species have been identified within this genus, with hosts ranging from birds, reptiles and fish to mammals, including companion animals such as dogs and cats. Several *Chlamydia* species are of significant public health concern, including the human pathogens *C. trachomatis* and *C. pneumoniae*, as well as the zoonotic *C. psittaci, C. abortus, C. caviae* and *C. felis*. Although human infections with *C. felis* are rare, sporadic zoonotic cases have been documented, underscoring the need for continued surveillance of this pathogen within the One Health framework (2).

*Chlamydia felis* is the primary species responsible for infections in cats, although other chlamydial species including *C. abortus* (29), *C. pneumoniae* (28), *C. psittaci* (26) and *C. suis* (23) have occasionally been reported. Compared to other members of the *Chlamydiaceae* family, *C. felis* exhibits relatively high host specificity; however, it has also been detected in dogs (29).

In domestic cats, *C. felis* infection is a common cause of conjunctivitis. Clinical signs typically include ocular discharge, conjunctival swelling, blepharospasm and redness of the third eyelid. In some animals, mild systemic signs such as fever or nasal discharge may also occur. Although the infection often resolves spontaneously, in untreated animals or those with compromised immunity it may become chronic. Pathogen shedding in ocular secretions can persist for weeks, and intermittent shedding for several months has been reported, increasing the risk of transmission from asymptomatic carriers (30).

Chlamydiae are obligate intracellular pathogens with a reduced and conserved genome, which hinders strain differentiation using classical molecular methods in the case of *C. felis* (7, 9). Standard tests such as real-time PCR allow pathogen detection but do not enable assessment of genetic variability. For more detailed molecular characterisation, variable fragments of certain genes are analysed, for example fragments of *omp*A, which encodes the major outer membrane protein. Despite the overall intraspecific genomic conservation of *C. felis, omp*A shows significant variability, allowing phylogenetic differentiation of strains and identification of potential geographic or host-specific variants.

In contrast to other countries, Poland lacks up-to-date epidemiological and molecular data on *C. felis* covering both owned and free-ranging cat populations. Globally, the prevalence of *C. felis* infection in clinically healthy domestic cats typically ranges from 0 to 10%, while in cats with conjunctivitis it ranges from 5.6% to 30.9% (4, 5, 11). In stray and shelter populations, the prevalence is markedly higher, reaching 35.7%–65.8% in some studies (3, 12, 35). Other studies have analysed risk factors such as age, sex, environment quality and co-infections, as well as employed genetic marker sequencing to assess strain variability (1, 3, 10, 11). In light of these data, the only molecular study conducted in Poland over a decade ago (25) appears insufficient. The lack of current epidemiological and genetic analyses across various populations, including free-ranging and shelter cats, limits opportunities for international comparison and a comprehensive understanding of the prevalence and diversity of *C. felis* strains in Poland.

In addition to pathogen detection and genetic characterisation, identifying age, sex, breed and living environment factors that predispose cats to infection is of considerable importance. These data are essential for understanding pathogen transmission dynamics and for developing effective prevention strategies. The existing reports on this topic are limited and often pertain to specific populations, leaving the need unmet for studies in diverse groups of cats.

This study aimed to estimate the prevalence of *Chlamydiaceae* in cats and confirm the involvement of *C. felis*, to explore associations between age, sex, breed, origin and ocular signs and infection prevalence using logistic regression, and to provide a preliminary appraisal of the genetic diversity of *C. felis* based on *omp*A sequencing. The findings are expected to support improved diagnostic awareness and preventive approaches in feline chlamydiosis, particularly in high-risk groups of young and purebred cats.

## Material and Methods

### Samples

Conjunctival swabs were collected from 156 cats presented to the Department and Clinic of Animal Surgery of the University of Life Sciences in Lublin between January 2023 and December 2024. Both clinically healthy cats and those presenting with conjunctivitis were recruited during routine veterinary visits in order to ensure population diversity and representativeness. One swab sample was collected from the right conjunctival sac and one from the left except in one instance of a single swab being obtained. In total, 311 ocular swabs were collected. A standardised questionnaire was completed for each animal to obtain information on health status, breed, age, origin and other relevant data. Detailed information on the animals examined is provided in Table 1.

**Table 1.** Summary of demographic and clinical factors characterising the cats of which the conjunctival sacs were swabbed for *Chlamydia felis* testing

		Number of tested cats
Sex	Male	86
	Female	70
	0–1	23
Age (years)	2–3	47
	4–7	32
	≥8	54
	European Shorthair	87
	British Shorthair/Longhair	16
	Devon Rex	7
	Mixed breed	19
	Sphynx	8
Breed	Maine Coon	4
	Ragdoll	5
	Persian	2
	Scottish Fold	4
	Exotic Shorthair	1
	Siberian	1
	Siamese	1
	Norwegian Forest	1
	Cattery	47
	Stray	43
	Shelter	14
Origin	Raised from birth by one owner	2
	Adoption	8
	Other	42
Evident conjunctivitis	Yes	81
	No	75

### Ethics statement

The study was performed in accordance with Polish law and with Directive 2010/63/EU of the European Parliament and of the Council of 22 September 2010 on the protection of animals used for scientific purposes, Chapter I, Article 1, point 5(b). Formal ethical approval was not required as conjunctival swabbing was performed during routine clinical procedures without experimental interventions. Written informed consent was obtained from all owners prior to sampling and study participation, and all data were anonymised before analysis.

### Isolation of DNA

Extraction of DNA from conjunctival swabs was carried out using the QIAamp DNA Mini Kit (Qiagen, Hilden, Germany) following the manufacturer’s instructions. An internal positive control (TaqMan Exogenous Internal Positive Control, Applied Biosystems, Foster City, CA, USA) was added to each sample according to the manufacturer’s protocol to distinguish true negative results from negatives which were false because of PCR inhibition. Extracts of DNA were stored at –20°C until analysis.

### Real-time PCR

A *Chlamydiaceae*-specific real-time PCR assay targeting a conserved 111-base-pair fragment of the 23S rRNA gene (8) was applied for initial screening. Species-specific real-time PCR assays were subsequently performed on all *Chlamydiaceae*-positive samples to identify *C. felis, C. abortus, C. pecorum* and *C. suis* (23); *C. gallinacea* (17); *C. psittaci* (21); and *C. avium* (36). All PCR reactions were run on a 7500 Real-Time PCR System (Applied Biosystems). Each assay included a panel of controls: species-specific positive control DNA and negative controls containing DNase/RNase-free water (Qiagen).

### Sequencing

Selected *C. felis*-positive samples (n = 13) obtained from 10 cats were used for amplification of part of the *omp*A (major outer membrane protein) gene. Specific fragments of *omp*A were amplified using CTU 5′-ATGAAAAAACTCTTGAAATCGG-3′ and CTL 5′-CAAGATTTTCTAGA(T/C)TTCAT(C/T)TTG-3′ (19) primer sets on a Biometra thermocycler (Göttingen, Germany). The products of the PCR were separated on 1% agarose gel stained with SimplySafe dye (EURx, Gdańsk, Poland). Amplicons were sequenced by Genomed (Warsaw, Poland).

### Phylogenetic analysis

Alignment of *omp*A sequences was created with MAFFT (multiple alignment using fast Fourier transform) (16) in Geneious Pro 8.0 (Biomatters, Auckland, New Zealand). Nucleotide identities were estimated from the resulting sequence alignment in Geneious Pro 8.0. A phylogenetic dendrogram was constructed using IQ-TREE v1.6.12 (22), applying 1,000 bootstrap replicates and selecting the best-fit model according to the Bayesian information criterion, and the tree was visualised using interactive Tree of Life (iTOL) v.7.2.1 (18). The model was selected with the ModelFinder module of IQ-TREE v1.6.12 (13, 15, 22, 32).

### Statistical analysis

Associations between infection status and demographic variables (age, sex, breed, origin and clinical signs) were initially assessed using odds ratios (OR) with 95% confidence intervals (CI) and P-values. In addition, an extended statistical analysis was performed to identify and quantify risk factors associated with *C. felis* infection by combining descriptive statistics with predictive modelling. All analyses were conducted in the Python programming environment using standard data-science libraries. The dataset comprising 156 cats was processed with the Pandas library (20) for data cleaning and standardisation. Categorical variables (*e.g*. breed and sex) were transformed into a numerical format using one-hot encoding, while continuous variables (*e.g*. age) were standardised to ensure comparability. These pre-processing steps were integrated into the Pipeline and ColumnTransformer Scikit-learn computational pipeline (24) to maintain consistency and prevent data leakage.

Exploratory data analysis was performed using Pandas, Matplotlib (14) and Seaborn (34) to calculate descriptive statistics (mean, median and standard deviation) and to visualise variable distributions and preliminary associations (histograms, boxplots and bar charts).

For predictive modelling, a logistic regression classifier (using Scikit-learn) was applied because it was suitable for binary outcomes (infected *vs* non-infected). Model performance was evaluated using accuracy, regression coefficients and odds ratios (OR) with 95% CI. The dataset was divided into training and test sets to ensure an objective assessment of predictive ability. In addition, Kaplan–Meier survival curves were generated to illustrate the probability of a cat remaining infection-free at different ages. This multi-step workflow enabled a robust evaluation of the impact of age, breed, sex and ocular disease on the likelihood of *C. felis* infection.

For statistical analysis, British Shorthair and British Longhair were considered jointly as a single category (British Shorthair/Longhair).

## Results

### Prevalence and real-time PCR results

Ocular swab testing by real-time PCR revealed that 12 out of 156 cats were infected with bacteria of the family *Chlamydiaceae*, corresponding to a prevalence of 7.7% (Table 2). All positive samples were identified exclusively as *C. felis*. No other *Chlamydia* species were detected.

**Table 2.** Prevalence data for *C. felis* in cats cross-referenced with demographic and clinical factors

		Number of tested cats	Number of *Chlamydiaceae/C. felis-*positive cats
Sex	Male	86	10
	Female	70	2
Age (years)	0–1	23	-
	2–3	47	8
	4–7	32	3
	≥8	54	1
Breed	European Shorthair	87	6
	British Short-/Longhair	16	3
	Devon Rex	7	2
	Mixed-breed	19	-
	Sphynx	8	-
	Maine Coon	4	-
	Ragdoll	5	-
	Persian	2	-
	Scottish Fold	4	-
	Exotic Shorthair	1	-
	Siberian	1	-
	Siamese	1	-
	Norwegian Forest	1	1
Origin	Cattery	47	5
	Stray	43	4
	Shelter	14	2
	Raised from birth by one owner	2	1
	Adoption	8	-
	Other	42	-
Evident	Yes	81	11
conjunctivitis	No	75	1

Threshold cycle (Ct) values for positive samples showed considerable variability (Table 3). In the *Chlamydiaceae*-specific assay, the mean C_t_ was 21.65 for the right eye and 20.64 for the left eye; in the *C. felis*-specific assay, the corresponding means were 21.49 and 17.19. Unilateral infection was observed in 8/12 positive cats; however, in one cat only a single eye was sampled, precluding definitive classification as unilateral infection, while bilateral involvement occurred in 4/12, indicating that localised ocular infections predominated.

**Table 3.** Demographic data and real-time PCR results (threshold cycle (C_t_) values) for *Chlamydiaceae-* and *C. felis*-positive cats from ocular swabs

Cat ID	Sex	Age (months)	Breed	Origin	*Chlamydiaceae*-positive C_t_		*C. felis*-positive Ct	
					Right eye	Left eye	Right eye	Left eye
23-10A(1/2)	M	36	British Longhair	Cattery	23.97	24.69	23.90	24.40
23-91A(1/2)	M	6	British Shorthair	Cattery	23.06	negative	22.91	negative
23-162A(1/2)	M	84	European Shorthair	Raised from birth by one owner	negative	35.47	negative	34.19
23-340A	M	7	European Shorthair	Shelter	27.1	not tested	26.90	not tested
23-393A(1/2)	M	18	British Shorthair	Cattery	31.23	negative	31.04	negative
23-445A(1/2)	M	30	Norwegian Forest	Shelter	negative	31.26	negative	30.84
23-716A(1/2)	M	24	Devon Rex	Cattery	26.09	negative	26.05	negative
24-1A (1/2)	M	12	European Shorthair	Stray	negative	25.66	negative	25.93
24-10A(1/2)	F	6	European Shorthair	Stray	31.98	32.83	32.13	32.65
24-101A(1/2)	M	48	European Shorthair	Stray	31.38	28.33	30.79	27.95
24-370A(1/2)	M	not available	Devon Rex	Cattery	35.77	33.91	35.51	33.33
24-491A(1/2)	F	6	European Shorthair	Stray	29.26	negative	25.56	negative

1 C_t_ – threshold cycle value in real-time PCR

### Demographic characteristics

The mean age of the 11 *C. felis*-positive cats with known age (of the total 12) was 2.5 years (95%, CI 1.41–3.59), and the median was 2 years, which were significantly lower than those of the overall study population (mean 5.04 years, median 3 years, range 0.17–18 years and standard deviation 4.56). Males predominated among infected individuals (10/12; 83.3%). More than half of the infected cats were purebred (7/12; 58.3%), mainly British Shorthair/Longhair and Norwegian Forest cats, and 5/12 (41.7%) were European Shorthair. Nearly half of the infected cats had come from catteries (5/12; 41.7%), strays were the next subgroup by size (4/12; 33.3%), shelter cats were a small fraction (2/12; 16.7%) and only a single cat was raised from birth by one owner (1/12; 8.3%) (Table 2). These group-level distributions are summarised in Table 2.

### Age-related risk factors

**Fig. 1:**
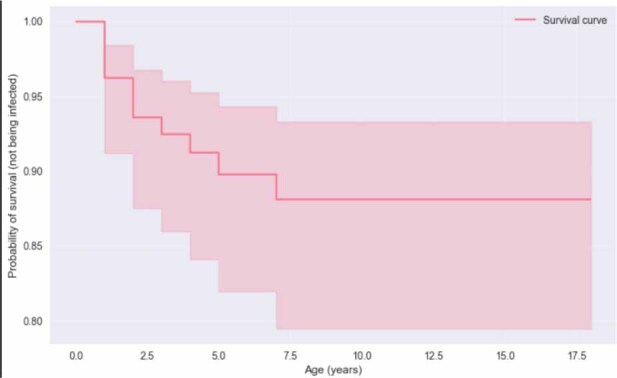
Kaplan–Meier survival curve showing the probability of remaining infection-free by age in cats

Logistic regression confirmed that age acted as a protective factor, yielding a regression coefficient of 0.1744, which indicated that each additional year of life reduced the probability of infection. The predictive accuracy of the model was high (93.5%). However, this value should be interpreted with caution given the limited number of positive cases, although the model still identified age, ocular signs and breed as the main predictors of infection. The Kaplan–Meier survival curve (Fig. 1) was used solely for illustrative purposes to visualise age-related differences in infection prevalence. As the study design was cross-sectional rather than longitudinal, the curve should be interpreted as exploratory, with the primary conclusions based on logistic regression analysis. The steepest decline in infection-free probability was observed during the first 2–4 years of life, while after approximately 7 years the risk of new infection appeared to plateau.

### Ocular health as an associated factor

Ocular health emerged as a critical associated factor, as conjunctivitis was a significant predictor of infection. Cats with ocular abnormalities were found to have nearly three times the odds of testing PCR positive (regression coefficient: 1.0562). This result provides strong evidence for the close association between *C. felis* and ocular disease.

### Breed-related risk factors

Breed analysis revealed variable susceptibility. The highest risk was observed in British Shorthair/Longhair cats, with a regression coefficient of 1.1975, corresponding to an OR of 3.31. This means that, after adjusting for other factors, British Shorthair/Longhair cats had more than three times higher odds of infection compared with European Shorthairs.

**Fig 2.**
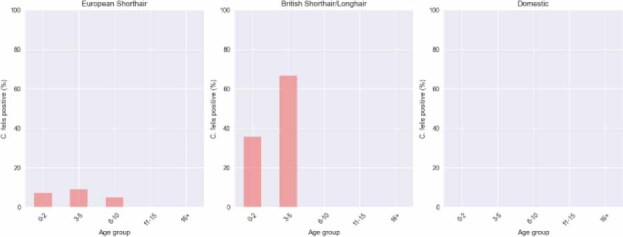
Prevalence of *C. felis* infection in cats by age group and breed

The prevalence of infection among British Shorthair/Longhair cats reached 40% in the 0–2 year age group and nearly 70% in the 3–5 year group, whereas in European Shorthair cats it did not exceed 10%, and in mixed-breed cats no infections were recorded. Interaction analysis between age and breed demonstrated that, although age generally acted as a protective factor, this decline in risk was markedly weaker in British Shorthair/Longhair (interaction OR of 1.65) and European Shorthair cats (interaction OR of 1.41). Consequently, these breeds remained at relatively high risk of infection for longer periods of their lives than other breeds. These differences are illustrated in a bar chart showing infection prevalence by age group and breed (Fig. 2). However, some age–breed subgroups included only a few animals, so these estimates should be interpreted with caution.

### Phylogenetic analysis

The partial *omp*A sequences from the present study were deposited in the GenBank (NCBI) database under accession Nos PX289947–PX289959.

**Fig 3.**
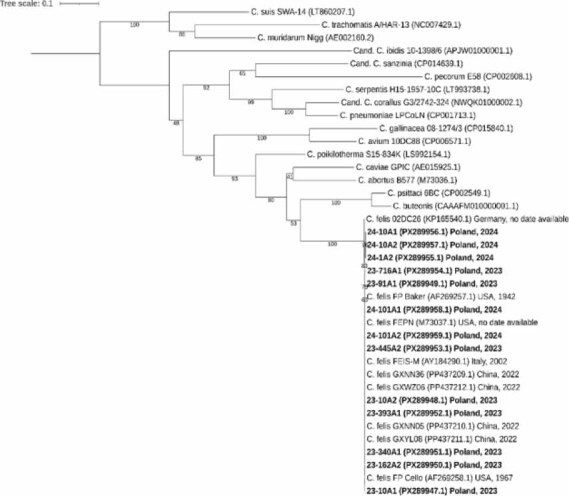
Analysis of the partial *omp*A gene of *C. felis* and representatives of *Chlamydia* spp. The phylogenetic tree is based on 808-base-pair consensus alignment and was constructed by the maximum-likelihood method with best-fit model according to the Bayesian information criterion general time reversible model with free base frequencies, invariant sites and γ four-rate category distribution, with support values calculated from 1,000 bootstraps. The scale bar represents the number of nucleotide substitutions per site. Sequences obtained in this study are marked in bold. GenBank accession numbers are shown in brackets

Sequence analysis of the *omp*A gene fragment revealed low genetic diversity among the obtained sequences (n = 13) and other strains available in GenBank, with nucleotide sequence identity ranging from 99.7% to 100%. Based on phylogenetic analysis, the *omp*A amplicons clustered into a single clade within the *C. felis* branch together with strains originating from the USA, Italy, Germany and China (Fig. 3).

## Discussion

The prevalence of *Chlamydiaceae* in the examined cat population was 7.7%, and all positive samples were identified as *C. felis*. This result falls within the ranges reported in other studies, where the proportion of *C. felis* infections varied from a few to several dozen percent, depending on geographic location, population characteristics and the diagnostic methods employed (3, 4, 5, 11, 12, 33). The data obtained represent the first study of this pathogen in cats in Poland in a long period, filling a gap left by earlier reports and enabling comparison with findings from other regions worldwide (25).

The absence of other *Chlamydiaceae* species may indicate that *C. felis* is the predominant – and potentially the only clinically relevant – member of this bacterial family affecting cats. Similar observations have been reported in studies conducted in other countries: in Switzerland, *C. felis* accounted for 92.2% of positive cases (with only 2.6% attributed to *C. abortus*) (3), and in the Szeged region of Hungary, PCR-based sequencing detected exclusively *C. felis* in all confirmed cases (33).

Our phylogenetic analysis confirmed the high conservation of the *omp*A gene, consistently with previous studies from other regions (7). This suggests that *C. felis* represents a globally homogeneous population (3, 33). Such genetic stability supports the applicability of *omp*A-based diagnostics across geographic settings and reduces the likelihood of emerging antigenic variants, although this requires confirmation in immunological studies.

The C_t_ values obtained in real-time PCR assays for *Chlamydiaceae* and *C. felis*, reflecting pathogen shedding and replication intensity in the eye, showed considerable variability, as evidenced by wide 95% CIs. This heterogeneity may result from differences in the stage of infection, host immune response or sample collection efficiency. An asymmetric distribution of C_t_ values and the presence of outliers were also observed in other studies on *C. felis*, where such variability was attributed to the heterogeneous course of the disease and differences in host immune responses (31). Co-infections with other feline respiratory pathogens, such as felid herpesvirus 1, feline calicivirus or *Mycoplasma felis*, may also influence the replication dynamics of *C. felis*. Damage to the conjunctival or corneal epithelium caused by viral or bacterial infections could facilitate *C. felis* proliferation, resulting in lower Ct values, whereas an active immune response or prior antibacterial treatment could suppress its replication, leading to higher Ct values. In the present study, however, the potential impact of co-infections can only be considered in the light of the existing literature, since no parallel diagnostics were performed.

*Chlamydia felis* infection was more frequently unilateral than bilateral among PCR-positive cats. This suggests that the infection may initially develop locally, with progression to both eyes depending on individual factors such as host immunity, pathogen exposure intensity or the presence of co-infections. Similar observations of unilateral infections have been reported in other studies, highlighting the clinical variability of feline chlamydiosis (27).

Analysis of demographic factors confirmed a higher risk in younger cats, which is consistent with previous studies linking susceptibility to immune system immaturity and environmental factors facilitating pathogen transmission (12, 30). The predominance of infected males may be attributed to their greater mobility and more frequent contact with other cats. Breed also emerged as a significant factor: British Shorthair/Longhair cats had more than three-times-higher odds of infection than European Shorthair cats, and their susceptibility persisted over a longer period of life. The absence of infections in mixed-breed cats may suggest either genetic predisposition in purebreds or environmental factors, such as high animal density in the breeding facilities from which purebreds come and which are not typically the origin of mixed-breed cats. The higher prevalence observed in purebred cats may reflect either breed-related susceptibility or environmental factors, such as higher animal density in breeding facilities.

Our analysis provides strong evidence that *Chlamydiaceae* represents a particular threat to young cats. Age acts as a natural protective factor, with infection risk steadily decreasing over subsequent years of life. From a clinical perspective, this means that diagnostic testing for chlamydiosis should be a priority in young patients, especially those presenting with ocular symptoms.

This study has some limitations. The relatively small number of positive cases limits the statistical power and generalisability of the findings. Although the logistic regression model achieved a high classification accuracy (93.5%), this value should be interpreted with caution because of the imbalance between positive and negative cases, which may have inflated the accuracy estimate. Similarly, the Kaplan–Meier analysis was applied primarily to illustrate trends in infection-free probability by age and should be regarded as exploratory rather than definitive. Additionally, material was collected from cats treated at a single veterinary clinic, which may limit the representativeness of the sample. Co-infections with other feline respiratory pathogens were not assessed and could have influenced both clinical presentation and Ct value variability. Despite these limitations, the model consistently highlighted age, ocular signs and breed as key predictors of infection, supporting the biological plausibility of the identified risk and associated factors.

## Conclusion

This study provides updated data on the occurrence of *C. felis* in cats and places it within a broader international context. The results demonstrate that infection is primarily identified in young animals presenting with ocular symptoms and that breed increases susceptibility, with British Shorthair/Longhair cats at particularly high risk. These findings underline the multifactorial nature of *C. felis* infection and highlight the need for prioritised diagnostic testing and preventive measures in groups with a predictor and high-risk groups. Future research including samples from multiple veterinary clinics and shelter populations would provide a broader and more representative epidemiological perspective on *C. felis* infections in cats.
